# Retinal Protection and Distribution of Curcumin *in Vitro* and *in Vivo*

**DOI:** 10.3389/fphar.2018.00670

**Published:** 2018-06-22

**Authors:** Chiara B. M. Platania, Annamaria Fidilio, Francesca Lazzara, Cateno Piazza, Federica Geraci, Giovanni Giurdanella, Gian Marco Leggio, Salvatore Salomone, Filippo Drago, Claudio Bucolo

**Affiliations:** ^1^Department of Biomedical and Biotechnological Sciences, School of Medicine, University of Catania, Catania, Italy; ^2^Unifarm, University of Catania, Catania, Italy; ^3^Center for Research in Ocular Pharmacology – CERFO, University of Catania, Catania, Italy

**Keywords:** curcumin, diabetic retinopathy, TNF-α, oxidative stress, reactive oxygen species

## Abstract

Diabetic retinopathy (DR), a secondary complication of diabetes, is a leading cause of irreversible blindness accounting for 5% of world blindness cases in working age. Oxidative stress and inflammation are considered causes of DR. Curcumin, a product with anti-oxidant and anti-inflammatory properties, is currently proposed as oral supplementation therapy for retinal degenerative diseases, including DR. In this study we predicted the pharmacodynamic profile of curcumin through an *in silico* approach. Furthermore, we tested the anti-oxidant and anti-inflammatory activity of curcumin on human retinal pigmented epithelial cells exposed to oxidative stress, human retinal endothelial and human retinal pericytes (HRPCs) cultured with high glucose. Because currently marketed curcumin nutraceutical products have not been so far evaluated for their ocular bioavailability; we assessed retinal distribution of curcumin, following oral administration, in rabbit eye. Curcumin (10 μM) decreased significantly (*p* < 0.01) ROS concentration and TNF-α release in retinal pigmented epithelial cells and retinal endothelial cells, respectively. The same curcumin concentration significantly (*p* < 0.01) protected retinal pericytes from high glucose damage as assessed by cell viability and LDH release. Among the tested formulations, only that containing a hydrophilic carrier provided therapeutic levels of curcumin in rabbit retina. In conclusion, our data suggest that curcumin, when properly formulated, may be of value in clinical practice to manage retinal diseases.

## Introduction

Curcumin (1,7-bis(4-hydroxy-3-methoxyphenyl)-1,6-heptadiene-3,5-dione) is an active ingredient extracted from the root of *Curcuma longa* showing anti-inflammatory and antioxidant properties, to be exploited for treatment of diabetes and also diabetic retinopathy (Jimenez-Osorio et al., 2015). Curcumin was found to be effective in inhibition of retinal vascular leakage in an animal model of diabetic retinopathy (DR) (Li et al., 2016). DR is one of the secondary complications of diabetes, and is included by the World Health Organization in the priority list of eye diseases, that can be partly prevented, but not cured yet. Duration of diabetes, glycemic control, blood pressure, response to insulin, nutritional and genetic factors can influence DR progression and burden. Several studies report that chronic inflammation comes along with DR leading to disease progression and worsening (Feenstra et al., 2013; Capitao and Soares, 2016). Furthermore, oxidative stress can have a detrimental role in establishment and progress of DR (Calderon et al., 2017). DR progresses from the non-proliferative phase (NPDR) to the proliferative phase (PDR). Generally, NPDR is asymptomatic, although retinal damage (microaneurysm) can be detected in NPDR patients by means of fundus analysis. Early/mild NPDR can progress to moderate/severe NPDR, characterized by retinal hemorrhages and diabetic macular edema (DME). When DR progresses to PDR with DME the risk of retinal detachment and irreversible blindness increases. Thus, diabetic patients need to undergo to early and frequent ophthalmologic examinations in order to monitor the development of retinopathy. Nowadays, there are no therapies of early DR and approved treatments are addressed to DME: photocoagulation laser therapy, intravitreal administration of corticosteroids and anti-VEGF drugs (Wykoff, 2017).

It is well known that oxidative stress is linked to inflammation, and the main sources of proinflammatory cytokines/chemokines in DR are locally synthetized by retinal pigmented epithelial (RPE) and glial cells (Bogdanov et al., 2017). Among inflammatory cytokines TNF-α has been showed to induce in RPE the upregulation of genes involved in apoptosis and cell morphology and migration, along to epithelial cell development (Korthagen et al., 2015). TNF-α can indeed damage the RPE cell layer, affecting the integrity of outer blood retinal barrier (Korthagen et al., 2015), that is strongly affected in diabetic retinopathy (Ved et al., 2017). Furthermore, TNF-α is up-regulated in the eyes of patients with diabetic retinopathy (Simo-Servat et al., 2016) and elicits reactive oxygen species (ROS) production and oxidative stress in human RPE cells (Yang et al., 2007). Furthermore, it has been recently demonstrated that TNFα up-regulates the expression of vascular endothelial growth factor (VEGF), a major angiogenic factor, in RPE cells via the ROS-dependent activation of β-catenin (Wang et al., 2016). RPE cells have been mainly used as *in vitro* model of age related macular degeneration (AMD), however, a series of reports have highlighted the involvement of RPE in diabetic retinopathy (Desjardins et al., 2016; Gonzalez de Vega and Garcia, 2018). Because DR is characterized by the breakdown of inner (microvascular endothelial and pericyte cells) and outer blood retinal barrier (RPE), to confirm the hypothesis that curcumin can exert antioxidant and anti-inflammatory activity in diabetic retinopathy, we tested curcumin on retinal pigmented epithelial cells (ARPE-19), human retinal endothelial cells (HRECs), and human retinal pericytes (HRPC) (Lupo et al., 2013; Giurdanella et al., 2015, 2017; Platania et al., 2017).

The pleiotropic action of curcumin (Di Martino et al., 2017) could be related to binding to a series of pharmacological targets other than COX-2, indeed, through an *in silico* approach we predicted other putative pharmacological targets of curcumin.

Therapeutic applications of curcumin can be limited by its poor water solubility, rapid hydrolysis in alkaline media and photodegradation, resulting in poor ADME (adsorption, degradation, metabolism, and excretion) properties (Jager et al., 2014).

Several efforts have been carried out in order to increase the oral bioavailability of curcumin, either by formulation with vehicles able to increase water solubility (soy lecithin, polyvinylpyrrolidone PVP) or by using compounds able to interfere with curcumin metabolism such as piperine (Jager et al., 2014).

Although curcumin is widely available in the market and used as nutritional supplement to protect visual function, its bioavailability in retinal tissue, after oral administration, remains to be assessed. We hypothesize that oral intake of curcumin may be of value in clinical practice to manage retinal diseases. Therefore, in order to demonstrate this hypothesis, we evaluated the curcumin distribution in rabbit retinal tissue after oral administration of commercial nutritional products. Furthermore, the pharmacological effective concentration of curcumin on human retinal cells exposed to oxidative stress or to high levels of glucose was assessed.

## Materials and Methods

### *In Silico* Study

**FIGURE 1:**
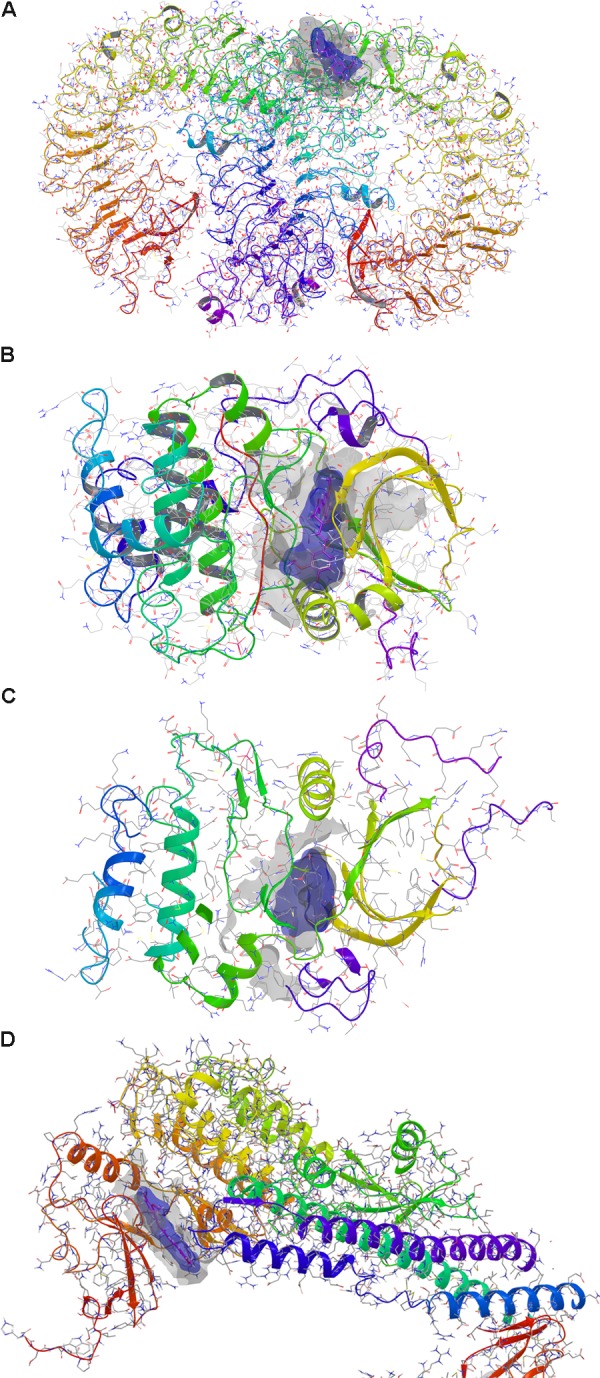
Docking of curcumin. **(A)** Toll-like receptor 9 dimer – extracellular region. **(B)** AKT1. **(C)** AKT2. **(D)** IκKβ. Gray solid surface indicates the receptor binding pocket; blue solid surface indicates curcumin Van der Waals surface.

Prediction of alternative pharmacological targets of curcumin was carried out by access to the webserver SwissTargetPrediction^1^ (Gfeller et al., 2014). SwissTargetPrediction generated as output a list of putative pharmacological targets of curcumin (**Table 1**). Based on previous reports about the binding of curcumin to Toll-like receptor (Youn et al., 2006) and Akt kinases (Jacot and Sherris, 2011; Chen et al., 2017; Fan et al., 2017), these two targets have been studied by means of molecular docking studies. Moreover, given the potential inhibitory effect of curcumin on TNF-α expression (Leclercq et al., 2004; Kowluru and Kanwar, 2007; Soetikno et al., 2011), we carried out molecular docking studies on IκKβ kinase, that when inhibited blocks the nuclear translocation of NFκB, a key transcription factor for TNF-α. The crystallographic structures (PDB codes) reported in **Table 2** were then retrieved with the protein preparation wizard of Schrodinger Maestro^®^; with this task ionization states were assigned to ionizable protein residues by setting pH at 7.4; then proteins were energy minimized with Prime^®^. Protein binding sites were detected with SiteMap^®^ task. Curcumin was docked to the selected molecular targets using the following docking protocol: (i) grid generation on the centroid of the binding pockets; (ii) standard precision (SP) docking (Schrödinger©) performed with Glide (Schrödinger©). First we carried out, docking with ring conformation sampling, followed by minimization (500 steps with conjugate gradient algorithm, dielectric = 1). Schrödinger© maestro was used to design the docking figures (**Figure 1** and **Supplementary Figure S1**).

**Table 1:** SwissTargetPrediction for curcumin.

Target	Uniprot ID	Gene code
Microtubule-associated protein tau	P10636	MAPT
Toll-like 9 receptor	Q9NR96	TLR9
Uncharacterized protein (by homology)	H0Y858	
Muscleblind-like protein 1	Q9NR56	MBNL1
Muscleblind-like protein 2 (by homology)	Q5VZF2	MBNL2
Muscleblind-like protein 3 (by homology)	Q9NUK0	MBNL3
Lactoylglutathione lyase	Q04760	GLO1
RAC-alpha serine/threonine-protein kinase	P31749	AKT1
RAC-beta serine/threonine-protein kinase (by homology)	P31751	AKT2
RAC-gamma serine/threonine protein kinase (by homology)	Q9Y243	AKT3
Testosterone 17-betadehydrogenase 3	P37058	HSD17B3
Estradiol 17-beta-dehydrogenase 12 (by homology)	Q53GQ0	HSD17B12
Quinone oxidoreductase (by homology)	Q08257	CRYZ
Gamma-secretase C-terminal fragment 59	P05067	APP

*Targets are listed in order of binding probability – from the highest to the lowest*.

**Table 2:** Molecular docking of curcumin.

Target	PDB code	Glide docking score
Toll-like receptor 9	3WPC	-3.899
AKT1	3CQW	-6.229
AKT2	2JDR	-9.818
IκKβ	4KIK	-7.973


### *In Vitro* Study

ARPE-19 (Retinal Pigment Epithelial immortalized cell line derived from Amy Aotaki-Keen eyes) cells were purchased by ATCC^®^ (Manassas, VA, United States). The human cell line was cultured at 37°C, in humidified atmosphere with 5% CO_2_. The DMEM:F12 Medium (ATCC No. 30-2006), including 100 U/ml penicillin 100 μg/ml streptomycin and 10% fetal bovine serum (FBS), was used for cell culture. Cell viability was assessed by measurement of ATP production by means of the ATPlite 1 step kit (Perkin Elmer 6016731, Monza, Italy) according to manufacturer’s protocol. ARPE-19 cells were plated into 96-well white plates (1.5 × 10^4^ cells per well). Cells were treated with increasing concentrations of curcumin (1–10–20–100 μM) for 24 h, in order to evaluate cell toxicity of curcumin (Tocris Bioscience, Bristol, United Kingdom). In another set of experiments, the protective effect of curcumin on ARPE-19 subjected to oxidative stress was evaluated as follows: 60 min treatment was carried out on ARPE-19 with 1–10–20–100 μM curcumin before induction of oxidative stress with 0.5 mM H_2_O_2_ treatment, carried out for 180 min. ATP levels in ARPE-19 were determined by measurements of luminescence with a Varioskan^TM^ Flash Multimode Reader (Thermo Fisher Scientific, Milan, Italy). ROS were measured by means of the DCFDA – Cellular Reactive Oxygen Species Detection Assay Kit (ab113851, Abcam Cambridge, United Kingdom), according to manufacturer’s protocol. ARPE-19 cells were plated into 96-well black plates (2 × 10 E4 cells per well), confluence was reached in 24 h, then cells were washed twice with phosphate-buffered saline (PBS pH 7.4) and incubated with 25 μM DCFDA in buffer solution at 37°C for 45 min. After two washes with PBS, cells were treated with increasing concentrations of curcumin (1–10–20–100 μM) for 60 min, then oxidative stress was induced with 0.5 mM H_2_O_2_ treatment for 120 min. ROS concentration was measured by detection of DCF fluorescence (λ_ex_ = 495 nm, λ_em_ = 529 nm) with Varioskan^TM^. HRECs were purchased from Innoprot^®^ (Derio – Bizkaia, Spain). Cells were cultured at 37°C, in humidified atmosphere (5% CO_2_), in Endothelial cell medium (ECM) supplemented with 5% FBS, 1% ECGS (Endothelial Cell Growth Supplement) and 100 U/ml penicillin 100 μg/ml streptomycin. HREC cells were plated into 60 mm × 15 mm Petri dishes (2 × 10^5^ cells per dish); after reaching 80% confluence, cells were shifted for 15 h to a low FBS medium (0.5% FBS). Cells were then cultured in medium (0.5% FBS) supplemented with physiological glucose concentration (5 mM) or with high levels of glucose (40 mM) (Huang et al., 2016). Pharmacological treatment of HREC was carried out with 10 μM curcumin in high glucose medium for 24 h. The concentration of curcumin, used for experiments on HREC cells, was chosen on the basis of dose-response curve of curcumin for ROS production inhibition. After treatments, the medium was collected in sterile tubes and centrifuged at 6,000 *g* for 10 min at 4°C; then supernatant was sampled. TNF-α concentration was measured by means of an ELISA kit (ADI-900-099, Enzo Life Sciences, Farmingdale, NY, United States) according to manufacturer’s protocol. HRPCs cells were purchased from INNOPROT. Cells were cultured at 37°C and in humidified atmosphere with 5% CO_2_. Cells were cultured in Innoprot-formulated Pericyte Medium, supplemented with 100 U/ml penicillin, 100 μg/ml streptomycin and 2% FBS till confluence (24 h). Therefore, cells were plated in 96-well plates (1 × 10^4^ cells) using a medium containing high glucose level (40 mM) and 10 μM curcumin. After 48 h cell-free culture supernatant was collected in empty plates and lactate dehydrogenase (LDH) release was measured using the Cytotoxicity Detection Kit^PLUS^ (LDH) (ROCHE 04744934001) according to manufacturer’s protocol. LDH was quantified by measuring absorbance at 490 nm with Varioskan^TM^ Flash Multimode Reader (Platania et al., 2017).

### *In Vivo* Study

Male New Zealand White rabbits (2.0–2.5 kg) were purchased from Envigo (Udine, Italy). Animals were housed under standard conditions, with free access to food and water, in a light-controlled (12-h light/12-h dark; lights on at 6 am) room at 21 ± 3°C and 54 ± 4% humidity. All rabbits were healthy and free of ocular abnormalities. Animal care and experimental procedures were carried out according to the Association for Research in Vision and Ophthalmology (ARVO) Statement for the Use of Animals in Ophthalmic and Vision Research and protocols approved by Institution Animal Care and Use Committee of the Catania University. The commercial products tested were: (a) curcumin formulation with a polyvinylpyrrolidone-hydrophilic carrier (CHC; Diabec^®^-AlfaIntes, Italy); (b) curcumin-phosphatidylcholine complex (CPC; Norflo^®^-EyePharma, Italy); (c) curcumin + piperine (CPI; AVS Retina^®^-SIFI, Italy). Before oral administration, two tablets of CPC and CPI were ground and quantitatively dissolved in sesame oil; two soft gel capsules of CHC were dissected and the content was quantitatively blended in sesame oil. The animals received single recommended (as indicated in the package leaflet of products) dose of curcumin formulations by oral gavage, after sedation (5 mg/kg Zoletil^®^ i.m.; Virbac, Milan, Italy) (*n* = 6 retinas for each time point).

After 0, 2, 6, 12, and 24 h from curcumin intake, the rabbits were sacrificed by intravenous administration of 0.3 ml/kg of Tanax^®^ (Intervet, Milan, Italy), after sedation (5 mg/kg of Zoletil^®^ i.m.; Virbac, Milan, Italy). Eye globes were enucleated *post mortem*, the retinas were collected, weighted and stored at -80°C till analysis.

### HPLC-MS/MS Analytical Method

Curcumin and salbutamol analytical standards were purchased from Sigma Aldrich (Milan, Italy). Curcumin was extracted from rabbit retina by methanol through the protein precipitation method, and salbutamol was used as the internal standard. Samples were weighted and homogenized with a grinder in ice-cold glass tubes. After adding methanol (150 μl) and salbutamol (35 ng/ml), the homogenates, were centrifuged for 15 min at 6,000 rpm at 4°C. The supernatant was transferred in a glass vial and then injected into LC-MS/MS for analysis. Sample analysis was carried out using an Agilent 6410 series triple quadrupole mass spectrometer with an electrospray ionization source (Agilent Technologies, Santa Clara, CA, United States). Chromatography was carried on a Phenomenex Kinetex C-18 analytical column (50 mm × 2.1 mm, 2.1 μm) with a HILIC for 2.1 mm guard cartridge (Phenomenex, Torrance, CA, United States). Isocratic elution was carried out with a mobile phase containing CH_3_CN (A) and 0.005% acetic acid in water (B), the ratio of the two solvents was set at 70:30 (A:B) v/v. Elution flow rate was set at 0.2 ml/min. Analytes were analyzed in positive ionization mode by using multiple reaction monitoring (MRM). The following ion transitions were monitored: 369 → 285 m/z and 240 → 148 m/z of curcumin and salbutamol, respectively (Ramalingam and Ko, 2014). Standard curve for curcumin was determined including control rabbit retinas as matrix; concentration range of standard curcumin was 0.93–46.87 ng/ml. Curcumin determination on each sample retina was carried out three times.

### Statistical Analysis

Operators were blind to treatments and samples analysis. All results were reported as mean ± SD. The results were analyzed using one-way ANOVA followed by Tukey–Kramer multiple comparisons test; differences between groups were considered significant for *p*-value < 0.05. *In vitro* results statistical analysis: mean ± SD were obtained from three independent experiments (three different cell plates). Furthermore, each cell treatment condition was replicated three times in each plate. Indeed, the sample size for *in vitro* study was *n* = 9. The results were analyzed using one-way ANOVA followed by Tukey–Kramer multiple comparisons test; differences between groups were considered significant for *p*-value < 0.05.

*In vivo* results statistical analysis: curcumin quantification was carried out for each formulation on 6 retinas per time-point (3 animals for time-point and 15 animals per formulation). Two-way ANOVA was carried out on data obtained from not repeated measures on each retina.

Graphs design and statistical analysis were carried out using GraphPad Prism 5 software (GraphPad Inc., San Diego, CA, United States).

## Results

### *In Silico* Study

Prediction of putative pharmacological targets of curcumin was carried out with SwissTargetPrediction. The output generated 15 pharmacological targets putatively modulated by curcumin (**Table 1**). Literature search was carried out in order to focus molecular docking on few targets that are involved in inflammatory response (**Table 2**); therefore, the binding mode of curcumin to these targets was predicted with molecular docking calculations (**Figure 1** and **Supplementary Figure S1**). In this respect, the most interesting targets that are involved in diabetic retinopathy and can bind curcumin were Toll-like 9 (Stewart et al., 2015; Jiang and Chen, 2017) receptor and Akt kinases (RAC serine/threonine protein kinase) (Jacot and Sherris, 2011). We also explored the binding of curcumin as IκKβ inhibitor. Indeed, we predicted the binding mode and calculated the docking score of curcumin bound to Toll-like receptor 9 dimer interface (extracellular region), AKT1-2 and IκKβ. Curcumin was predicted to bind Toll-like receptor 9 at the dimer interface, likely interfering with receptor dimerization and activation (**Figure 1A**). This result is in accordance to previous findings on curcumin binding at Toll-like receptor 4 (Youn et al., 2006). Furthermore, binding of curcumin into the inhibitor binding pocket of AKT1 and AKT2 (**Figures 1B,C**) was more favorable (more negative docking score) than binding at Toll-like 9 receptor dimer interface (**Table 2**). Indeed, curcumin may inhibit PI3K/AKT signaling pathway by acting as AKT inhibitor (Jacot and Sherris, 2011). Because TNF-α transcription is known to be controlled by NFκB, which, in turn, is activated following dissociation from phosphorylated IκKβ. Indeed, inhibition of IκB kinase (IκK) by curcumin, if occurring, would decrease NFκB transcriptional activity and TNF-α expression (Cavaleri, 2018). The docking score of curcumin bound to IκKβ (**Table 2** and **Figure 1D**) was more favorable than docking at AKT1 and Toll-like receptor 9. Thus, curcumin was predicted as able to modulate inflammatory cytokines expression by inhibition of IκKβ/NFκB signaling.

### *In Vitro* Study

**FIGURE 2:**
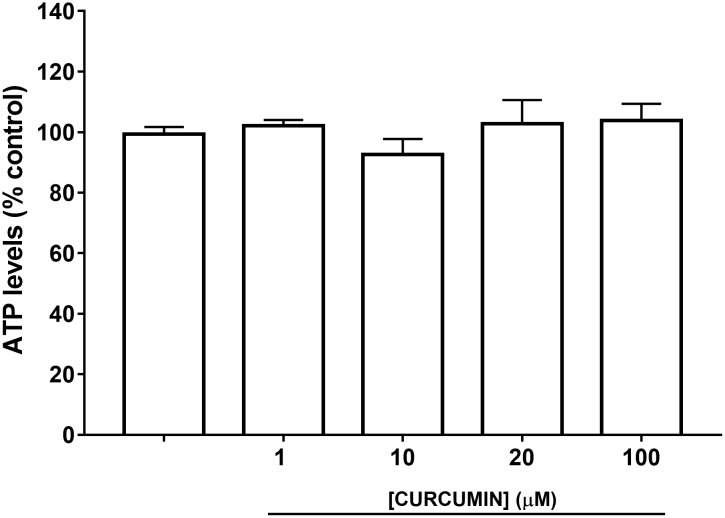
ARPE-19 cell viability.

**FIGURE 3:**
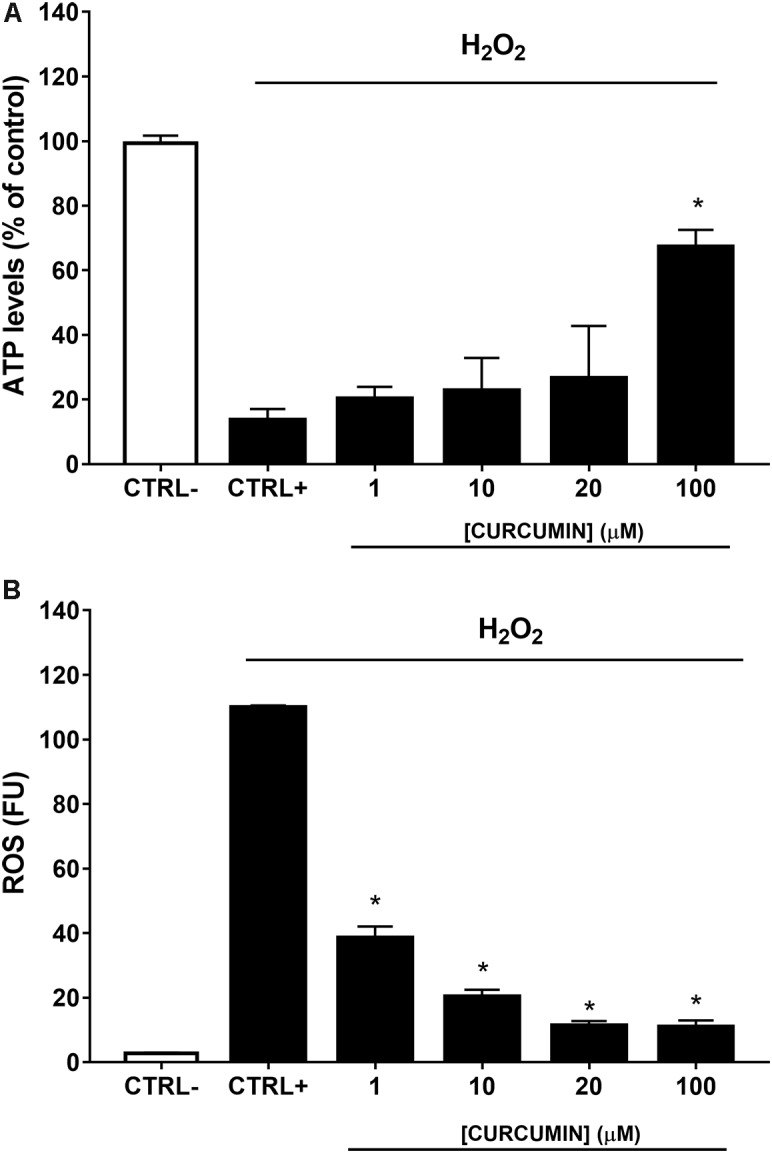
Curcumin protects ARPE-19 from oxidative damage. **(A)** Curcumin increased significantly cell viability of ARPE-19 exposed to oxidative stress. **(B)** Curcumin decreased ROS, expressed as fluorescent units (FU), production in ARPE-19, challenged with 0.5 mM H_2_O_2_. ^∗^*p* < 0.01 vs. CTRL+ (cells treated with H_2_O_2_).

A safety profile of curcumin, at different concentrations (1, 10, 20, and 100 μM), was assessed on ARPE-19 cells. Curcumin did not affect ARPE-19 cell viability (**Figure 2**) at all tested concentrations. Oxidative stress is known to be associated with diabetic retinopathy, while hyperglycemia induces the production of mitochondrial ROS (Calderon et al., 2017). Therefore, we used H_2_O_2_ to elicit oxidative damage in ARPE-19; 0.5 mM H_2_O_2_ exposure significantly reduced (*p* < 0.01) ARPE-19 viability; cell damage was attenuated, in a dose-dependent manner, by curcumin treatment (**Figure 3A**), even though only the highest concentration of curcumin (100 μM) reached a statistically significant effect (**Figure 3A**). Treatment with H_2_O_2_ also increased cellular ROS concentration (**Figure 3B**). Curcumin treatment, even at the lowest tested concentration (1 μM), significantly decreased (*p* < 0.01) cellular ROS production in ARPE-19, challenged with H_2_O_2_ (**Figure 3B**). The lowest ROS levels were detected following treatment with 20 μM curcumin on ARPE-19, subjected to oxidative stress. Furthermore, the effect of curcumin on ROS was concentration-dependent (EC_50_ 9.77 μM, *r*^2^= 0.9635 for non-linear fit “log [curcumin] vs. response – variable slope”).

**FIGURE 4:**
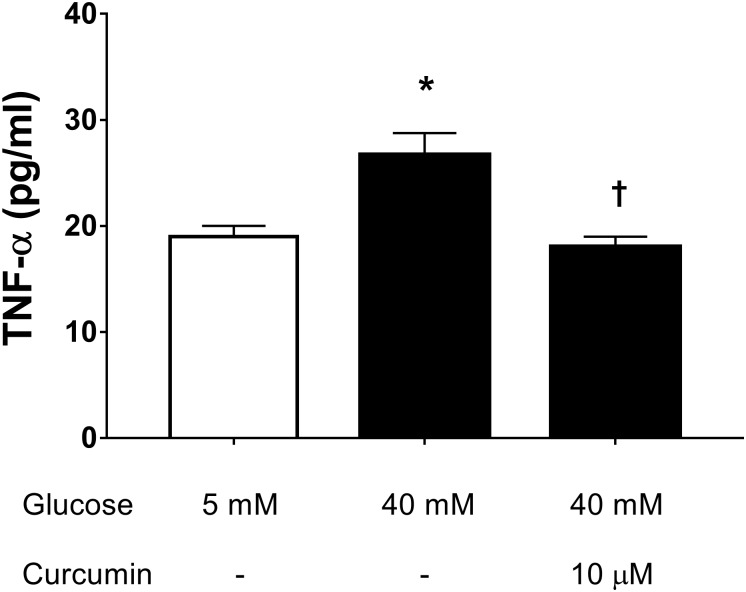
Curcumin decreases TNF-α in HREC exposed to in high glucose. ^∗^*p* < 0.01 vs. control group: cells cultured in physiological conditions of glucose (5 mM); ^†^*p* < 0.01 vs. cells cultured in high glucose (40 mM).

**FIGURE 5:**
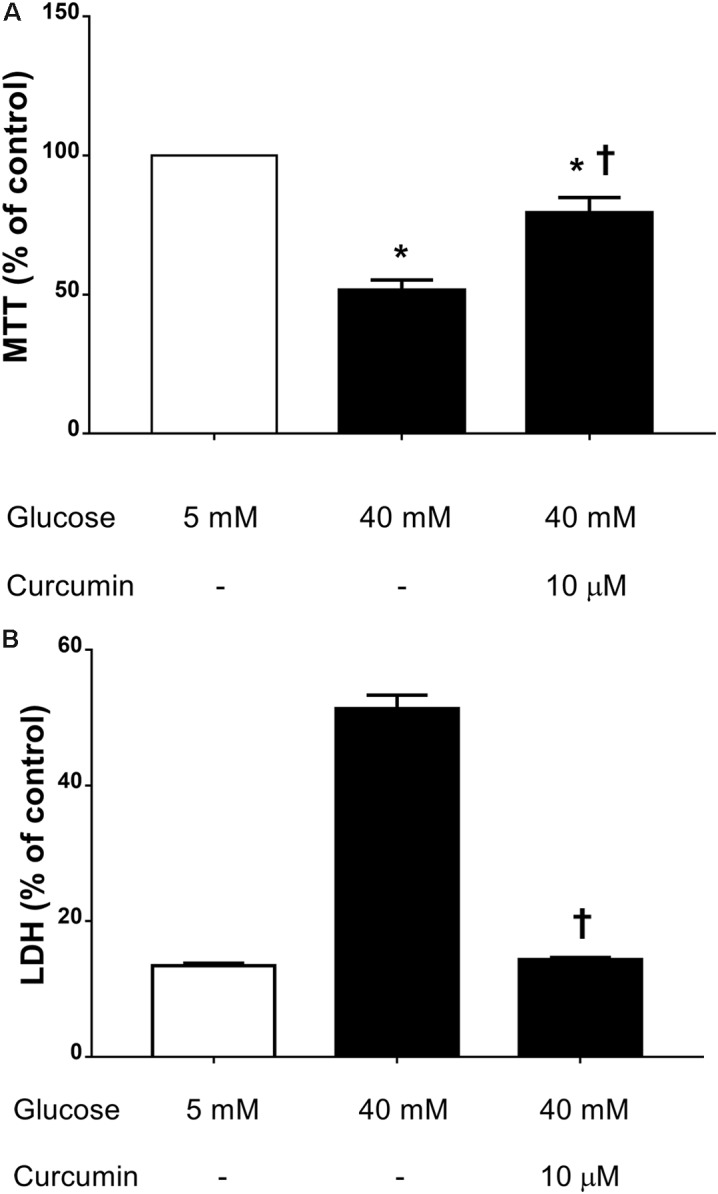
Curcumin increases HRPC viability and decreases cell death induced by high glucose. **(A)** MTT assay. **(B)** LDH release. ^∗^*p* < 0.01 vs. control group: cells cultured in physiological conditions of glucose (5 mM); ^†^*p* < 0.01 vs. cells cultured in high glucose (40 mM).

We then assessed the effect of 10 μM curcumin on TNF-α production in HREC cultured in medium with high glucose levels (40 mM) (Huang et al., 2016). We have chosen to test 10 μM curcumin on the basis of EC_50_ in ROS inhibition studies on ARPE-19. High glucose concentrations (40 mM) led to significant (*p* < 0.05) increase of TNF-α secretion by HREC (**Figure 4**). Treatment with 10 μM curcumin reverted the effect of 40 mM glucose leading to a significant decrease (*p* < 0.01) of TNF-α, that reached levels similar to those measured in control cells (5 mM glucose, **Figure 4**). Furthermore, we tested curcumin (10 μM) on HRPCs exposed to high glucose levels (40 mM). Curcumin (10 μM) significantly (*p* < 0.01) protected HRPC from the damage elicited by high glucose (**Figure 5A**). Furthermore, pericytes death induced by high glucose, was significantly decreased (*p* < 0.01) with 10 μM curcumin treatment (**Figure 5B**).

### *In Vivo* Study

**FIGURE 6:**
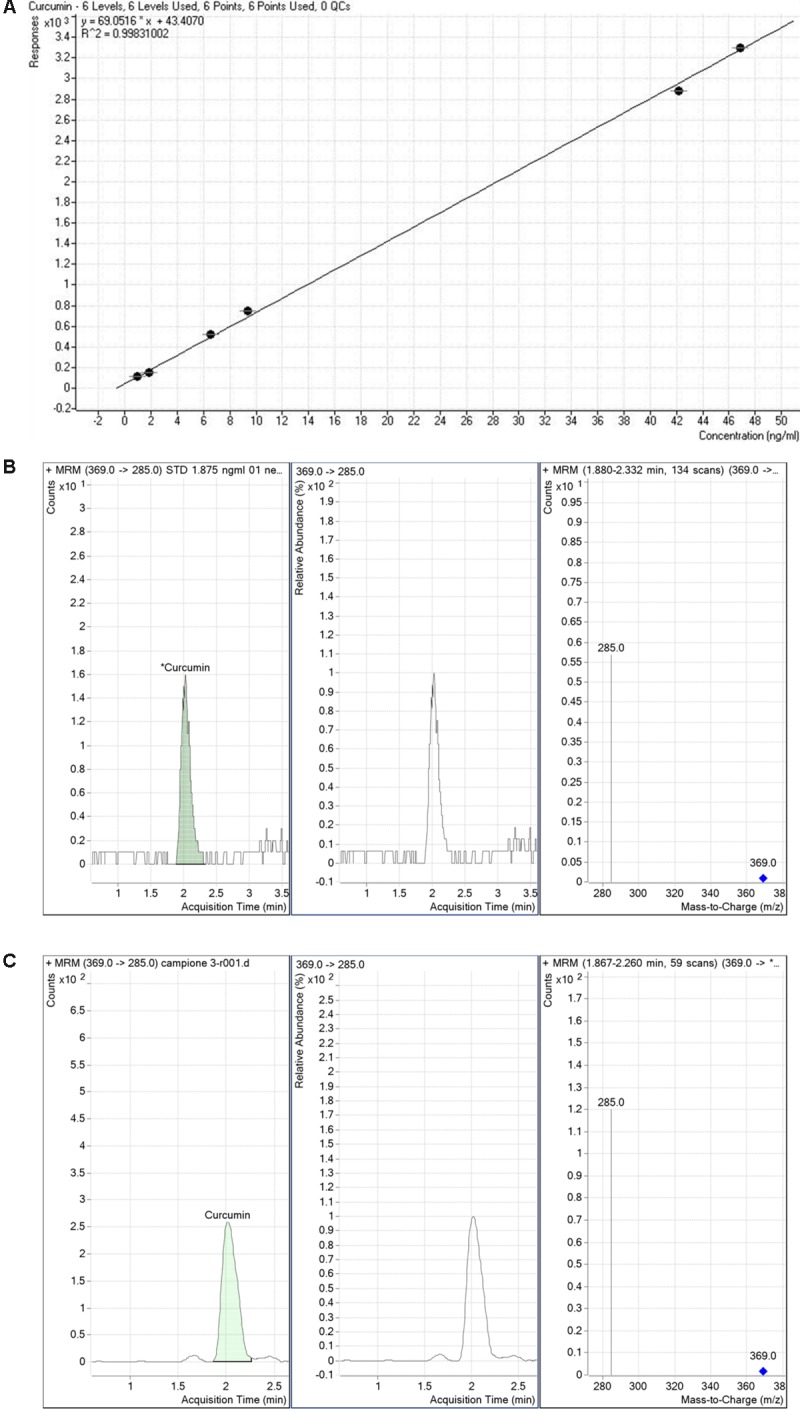
Detection of curcumin. Standard curve of curcumin **(A)**. Curcumin standard detection **(B)**. Representative peak **(C)** of curcumin detected in a rabbit retinal sample collected from polyvinylpyrrolidone-hydrophilic carrier (CHC) group.

**FIGURE 7:**
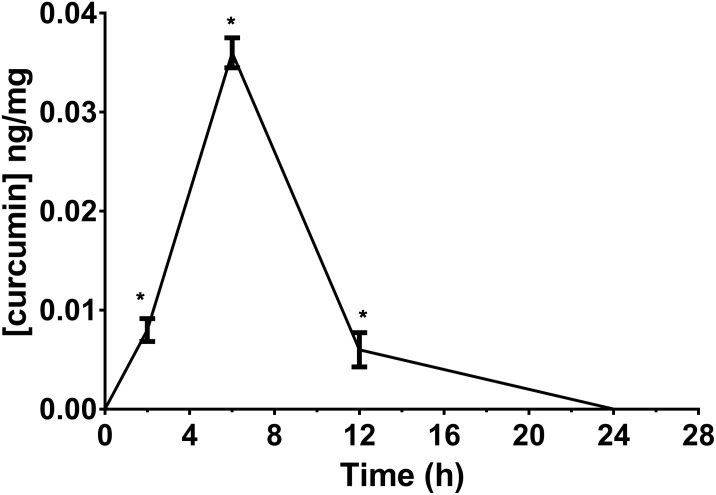
Retinal bioavailability of curcumin by oral route in rabbit. Curcumin formulation with a polyvinylpyrrolidone-hydrophilic carrier (CHC). ^∗^*p* < 0.01 vs. time 0 and control animals.

We determined the retinal bioavailability of curcumin after oral administration of commercial curcumin nutraceutical products (CHC, CPC, and CPI) to rabbits. Retinal distribution of curcumin was assessed through HPLC-MS/MS quantitative determination of curcumin, at different time points after oral administration. The *r*^2^ of standard curve of curcumin was 0.99831 (**Figure 6A**). **Figure 6B** shows the 285 m/z peak of standard curcumin, which was added to retinas of control rabbits (duplicated measurements). **Figure 6C** shows the peak of curcumin in the retina of a rabbit treated with CHC. Only curcumin administered as CHC formulation (Diabec^®^) was able to reach the rabbit retina (**Figure 7**). The *C*_max_ of curcumin (CHC) was 0.036 ± 0.002 ng per mg of retinal tissue, *T*_max_ was 6 h after oral administration of two capsules of CHC, and AUC_0-24h_ was 15.48 ± 0.05 ng^∗^min/mg (**Table 3**). Therefore, assuming a 70 mg average retinal weight, the overall retinal AUC of curcumin was 1.08 μg^∗^min. This amount of curcumin, as calculated from the retinal distribution of curcumin, is comparable to the amount used in the *in vitro* study; because, 10 μM curcumin concentration corresponds to 0.737 μg (200 μl per well, MW curcumin 368.38 Da).

**Table 3:** Retinal distribution of curcumin after oral administration.

Curcumin formulation	*C*_max_ ng/mg	*T*_max_	AUC_0-24__h_ ng^∗^min/mg
CHC	0.036 ± 0.002	6 h	15.48 ± 0.05
CPC	N.D.	N.A	N.A.
CPI	N.D.	N.A	N.A.


*N.D., not detectable; N.A., not applicable.*

## Discussion

Several studies reported that curcumin has anti-inflammatory and antioxidant activity, to be exploited for treatment of several diseases (e.g., atherosclerosis, Alzheimer disease, cancer, colitis) (Liu et al., 2016; Zhao et al., 2016; Cao et al., 2017; Kumar et al., 2017), including, those affecting the anterior segment of the eye (Liu et al., 2017). Preclinical studies showed that curcumin has a high therapeutic potential for treatment of retinal degenerations, such diabetic retinopathy, bearing chronic retinal inflammation, microvasculopathy, and oxidative stress as hallmarks (Feenstra et al., 2013; Calderon et al., 2017). Nutraceutical supplements are commonly used in clinical practice for treatment of retinal degeneration; such supplements generally include vitamin C, vitamin E, carotenoids, zinc, copper, ω-3, and polyunsaturated fatty acids (Buschini et al., 2015; Evans and Lawrenson, 2017). Worthy of note, curcumin supplementation is currently under clinical evaluation for treatment of diabetic retinopathy [NCT02984813, NCT01646047^2^].

The pleiotropic action of curcumin can derive from binding at several targets. In fact, curcumin has shown anti-inflammatory and anti-oxidant properties, related to inhibition of COX-2 (Zhang et al., 1999; Maldonado-Rojas and Olivero-Verbel, 2011) and induction of hemeoxygenase-1 by ARE/Nrf2 pathway (Scapagnini et al., 2011), respectively. Activation of Nrf2 pathway occurs with inhibition of the Keap1/Nrf2 protein-protein interaction. Nrf2 inducers act by: (i) disrupting Keap1-DC domain/Nfr2 interaction; (ii) covalent binding to cysteine residues of Keap1-BTB domain (Pittalà et al., 2017). Curcumin was predicted to induce hemeoxygenase-1 by preferential binding to Keap1-DC domain (Pittalà et al., 2017). On the basis of literature search Toll-like receptor 9, AKT 1–2, and IκKβ/NFκB were selected from the list of putative targets of curcumin, predicted by SwissTargetPrediction. In fact, previous experimental data showed that curcumin was able to modulate activity of these targets involved in inflammatory processes (Leclercq et al., 2004; Youn et al., 2006; Jacot and Sherris, 2011; Soetikno et al., 2011; Chen et al., 2017; Fan et al., 2017; Cavaleri, 2018). Therefore, we predicted through molecular docking calculations the binding mode of curcumin as inhibitor of the Toll-like receptor 9, of PI3K/AKT, and of the IκKβ/NFκB signaling pathways.

Furthermore, we found that curcumin is effective in *in vitro* models (retinal pigmented epithelial cells, retinal endothelial cells and pericytes) that recapitulate oxidative stress and inflammation in the diabetic retina (Rosales et al., 2014; Huang et al., 2016; Ved et al., 2017). The pharmacological effective curcumin concentrations used in our paradigms were superimposable to curcumin concentrations (5–20 μM) previously tested by other labs in *in vitro* models of retinal damage (Woo et al., 2012; Howell et al., 2013; Li et al., 2013).

Products containing curcumin are currently on the market claiming protective effects on retinal tissue; however, there are no data on retinal bioavailability of curcumin after oral intake of these formulations. Recently, curcumin plasma bioavailability has been evaluated, and the CHC formulation showed significant higher curcumin plasma levels (Jager et al., 2014), compared to either curcumin extract or curcumin-phosphatidylcholine complex. We hereby compared for the first time retinal curcumin retinal distribution of three nutraceutical products, indicated for retinal conditions, available in the market. A single recommended daily dose of CHC, CPC, and CPI formulations was administered to rabbits. We have found that only CHC formulation provided detectable retinal curcumin levels in comparison to CPC and CPI products, whose retinal levels were below the limit of calibration curve. The amount of curcumin that reached the rabbit retina, after CHC oral intake, was 1.08 μg^∗^min (AUC_0-24h_ 15.48 ng^∗^min/mg, retinal average weight = 70 mg). This amount is comparable to the effective pharmacological concentration of curcumin assessed by *in vitro* studies: (i) 0.720 μg curcumin (= 9.77 μM) that is the EC_50_ to decrease ROS in ARPE-19 cells; (ii) 7.37 μg (= 100 μM) that significantly increased ARPE-19 cell viability, (iii) 0.737 μg (= 10 μM) that significantly (*p* < 0.01) decreased TNF-α levels in HREC exposed to high glucose; (iv) 0.737 μg (= 10 μM) of curcumin that significantly (*p* < 0.01) protected pericytes form the damage elicited by high glucose.

Thus, CHC formulation is able to deliver retinal curcumin levels that are pharmacological effective in our *in vitro* paradigms. Finally, the present findings suggest that curcumin formulation with a polyvinylpyrrolidone-hydrophilic carrier may be of value in clinical practice to manage retinal diseases.

## Ethics Statement

Animal procedures followed guidelines of the Animal Care and Use Committee of the University of Catania and The Association for Research in Vision and Ophthalmology (ARVO) Statement for the Use of Animals in Ophthalmic and Vision Research.

## Author Contributions

CB and CBMP made substantial contributions to conception, design, and interpretation of data. CBMP, CP, FG, AF, GG, and GL analyzed and acquired the data. CB, CBMP, CP, FG, AF, GL, SS, and FD participated in drafting the article or revising it critically for important intellectual content and gave final approval of the version to be submitted and any revised version.

## Conflict of Interest Statement

The authors declare that the research was conducted in the absence of any commercial or financial relationships that could be construed as a potential conflict of interest. The reviewer EG and handling Editor declared their shared affiliation.

## Footnotes

1 http://www.swisstargetprediction.ch/

2 http://www.clinicaltrials.gov

## Supplementary Material

FIGURE S1 2D representation of docking poses of curcumin at binding pocket of **(A)** Toll-like receptor 9, **(B)** AKT1, **(C)** AKT2, and **(D)** IκKβ.
