# Supplementary figures and images for: Retinal Protection and Distribution of Curcumin in Vitro and in Vivo
Source: Front Pharmacol. 2018 Jun 22;9:670. doi: 10.3389/fphar.2018.00670 (PMC6036289; doi:10.3389/fphar.2018.00670)

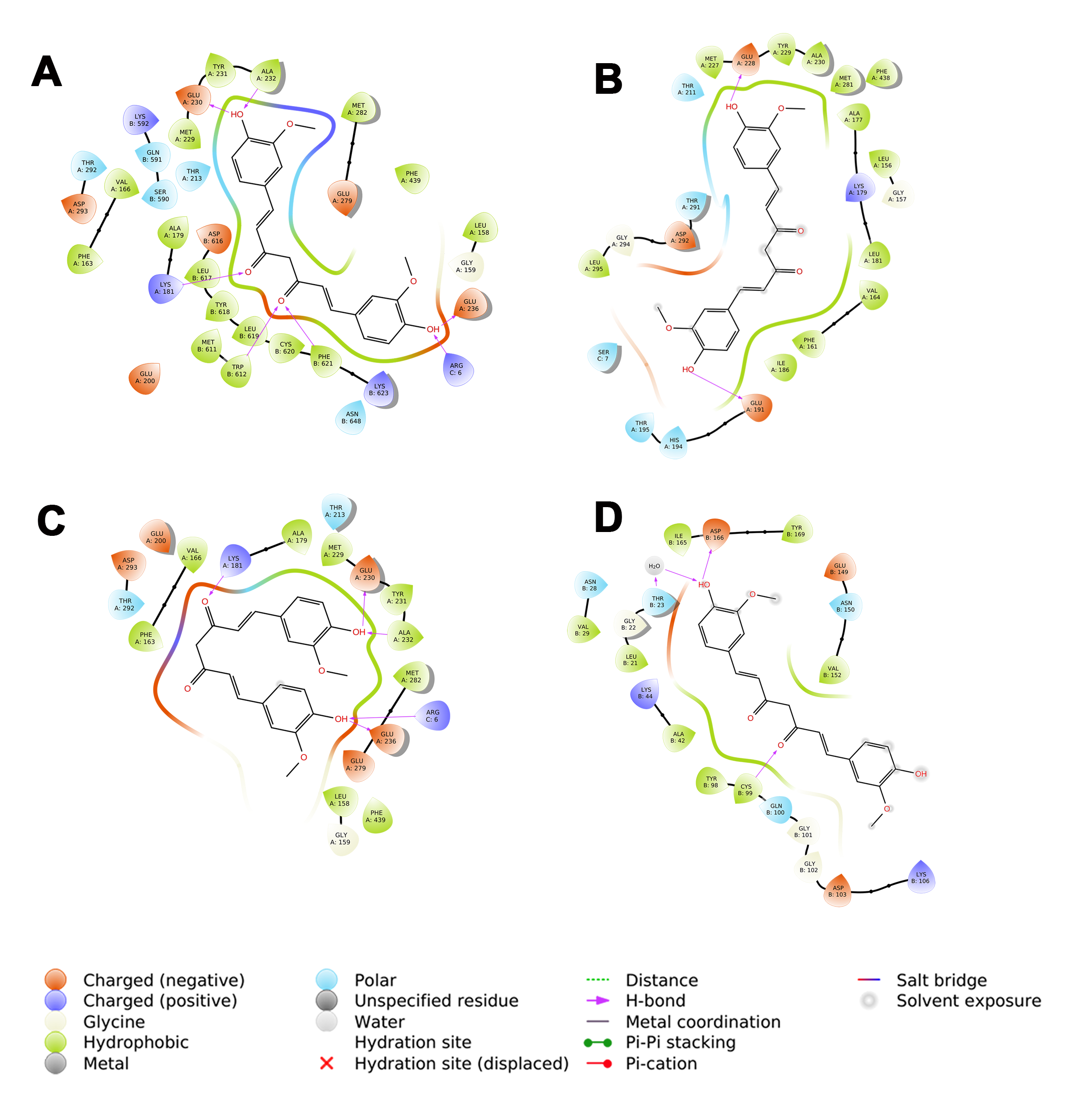

Supplement: FIGURE S1 — 2D representation of docking poses of curcumin at binding pocket of (A) Toll-like receptor 9, (B) AKT1, (C) AKT2, and (D) IκKβ. [file Image_1.TIF]
